# What goes on when the lights go off? Using machine learning techniques to characterize a child’s settling down period

**DOI:** 10.3389/fnetp.2025.1519407

**Published:** 2025-05-30

**Authors:** Deniz Kocanaogullari, Murat Akcakaya, Roxanna Bendixen, Adriane M. Soehner, Amy G. Hartman

**Affiliations:** ^1^ Swanson School of Engineering at the University of Pittsburgh, Pittsburgh, PA, United States; ^2^ Department of Occupational Therapy, Pittsburgh, PA, United States; ^3^ Department of Psychiatry at the University of Pittsburgh, Pittsburgh, PA, United States

**Keywords:** actigraphy, sleep, sleep onset delay, children, machine learning, sensory sensitivity, sensory processing, settling down

## Abstract

**Objectives:**

Current approaches to objective measurement of sleep disturbances in children overlook the period prior to sleep, or the settling down time. Using machine learning techniques, we identified key features that characterize differences in activity during the settling down period that differentiate children with sensory sensitivities to tactile input (SS) and children without sensitivities (NSS).

**Methods:**

Actigraphy data were collected from children with SS (n = 17) and children with NSS (n = 18) over 2 weeks (a total of 430 evenings). The settling down period, indicated using caregiver report and actigraphy indices, was isolated each evening and seven features (mean magnitude, maximum magnitude, kurtosis, skewness, Shannon entropy, standard deviation, and interquartile range) were extracted. 10-fold cross-validation with random forests were used to determine accuracy, sensitivity, and specificity of differentiating groups.

**Results:**

We could accurately differentiate groups (accuracy = 83%, specificity = 83%, sensitivity = 84%). Feature importance maps identify that children with SS have higher maximum bouts of activity (U = −2.23, p = 0.026) during the settling down time and a higher variance in activity for the children with SS (e.g., interquartile range, Shannon entropy) that sets them apart from their peers.

**Conclusion:**

We present a novel use of machine learning techniques that successfully uncovered differentiating features within the settling down period for our groups. These differences have been difficult to capture using standard sleep and rest-activity metrics. Our data suggests that activity during the settling down period may be a unique target for future research for children with SS.

## 1 Introduction

Actigraphy is a well-established method used to behaviorally assess sleep and rest-activity patterns across the lifespan. Wrist actigraphy uses a wrist-worn accelerometer to capture movement and infer sleep using sleep-wake algorithms. Actigraphy provides a home-based and less invasive option in sleep measurement compared to the gold standard polysomnography (PSG) (Smith et al., 2018). This is especially useful when examining sleep in pediatric populations who would find a PSG, conducted in a lab setting, invasive and disruptive.

Sleep health is a multidimensional characterization of sleep including sleep satisfaction, daytime alertness, sleep timing, efficiency, and duration (Buysse, 2014). While not all dimensions can be characterized by actigraphy, sleep timing, efficiency, and duration have been validated against PSG and normative values have been collected across multiple pediatric populations (Galland et al., 2018; Lamprecht et al., 2015; Meltzer et al., 2016; Ziegler et al., 2021; Yavuz-Kodat et al., 2019). These variables provide an important characterization of a child’s sleep period, from the moment of sleep onset to the last awakening in the morning.

In recent literature, *sleep behaviors* have been proposed to be an additional and highly relevant dimension for pediatric populations (Meltzer et al., 2021). Sleep behaviors include bedtime routine, parent-child interactions at bedtime, and bedtime timing. While actigraphy derived variables like sleep onset latency, or the time it takes to fall asleep, can give information about some sleep behaviors, we were interested in examining actigraphy data during the settling down period, a time after the bedtime routine during which a child is preparing to sleep.

Sensory sensitivities are prevalent for individuals across many neurodiverse groups (e.g., autism, attention-deficit hyperactivity disorder). Interestingly, neurodiverse groups with high rates of sensory sensitivities also report higher rates of sleep problems. Research has hypothesized that this could be related to differences in gating in the thalamus, sympathetic nervous system activation Our previous work suggests that children with sensory sensitivities (SS), specifically sensitivities to touch, report significantly higher rates of sleep difficulties using questionnaires compared to peers without sensitivities (NSS) (Hartman et al., 2022). However, when examining traditional sleep period (e.g., efficiency, duration) and rest-activity rhythm variables (e.g., daily variability in activity and rest), the only evident difference between children with SS and NSS was the average time it took for the children to settle down and fall asleep per caregiver report. We found that children with SS took an average of 30 min longer to settle down prior to sleep than their NSS peers (Hartman et al., 2023).

Our goal in this secondary analysis was to use machine learning techniques to analyze wrist actigraphy data from the settling down period in more detail to identify specifically how children with SS differ from their peers with NSS. Machine learning methodologies are powerful tools for analysis of time series data like sleep actigraphy data (Tilmanne et al., 2009). Current research uses machine learning techniques for sleep-wake identification (Cho et al., 2019; Rahikkala et al., 2019; Khademi, 2018; Khademi et al., 2019), sleep quality estimation (Bitkina et al., 2022), insomnia analysis (Rani et al., 2022) and isolated rapid-eye-movement sleep behavior disorder detection (Brink-Kjaer et al., 2022).

By applying machine learning techniques, we aimed to create and test a reliable, fast way to analyze the settling down period using actigraphy and sleep diary data. We aimed to add to the ways sleep behaviors might be measured in future studies. We hypothesize that there are unique aspects of the settling down period that contribute to the higher levels of reported sleep disturbances for children with SS. Understanding what goes on when the “lights go off” and children attempt to settle down to fall asleep will guide future research for interventions in this unique group of children.

## 2 Methods

This secondary data analysis uses actigraphy and sleep diary data from an observational study which collected data across a two-week period. All procedures for this initial study were approved by the University of Pittsburgh’s Institutional Review Board (STUDY20050082). All analyses were done using Python and its libraries pandas (McKinney, 2010) and scikit-learn (Pedregosa, 2011).

### 2.1 Participants

Children (6–10 years) and their caregivers were recruited across the United States using flyers shared over social media, listservs, and through the Pitt + Me research registry. Children were required to be English speaking and without a significant behavior or sleep diagnosis that impact their daily life (per caregiver report). The caregiver was required to complete daily sleep diary questions during their involvement of the study and report regular involvement in their child’s bedtime routine (at least four nights a week). Participants completed informed consent and data were collected between September and December 2021 (COVID-19 pandemic, Delta variant prominence).

The aim of the original study was to examine differences in sleep health for children with SS compared to peers with NSS. Therefore, two groups of children were recruited. Participants were included in the SS group if their parent endorsed 5+/7 screening questions [based off the Sensory Profile-2 (Dunn, 2014) tactile/oral tactile sensitivity questions; provided as Supplementary Material in previous manuscript (Hartman et al., 2022)]. Additionally, a diagnosis of autism, attention-deficit hyperactivity disorder, or Down’s syndrome excluded participants in both groups, as these diagnoses have different neurological and medical components beyond sensory sensitivities that may impact sleep. A demographics survey was utilized to record key variables and describe participants.

Demographics of the whole sample are reported in our previous paper (Hartman et al., 2022). For this secondary analysis, a total of 35 children were able to complete the data collection protocol with sufficient data for this analysis (at least four evenings of settling down time data from actigraphy and sleep diary): 17 children with SS and 18 with NSS (Table 1). The average age in both groups was around 7.7 years. The SS group was majority male (65%) and white, non-Hispanic (59%). The NSS group was majority female (61%) and white, non-Hispanic (89%).

**TABLE 1:** Participant demographics.

	Sensory Sensitive Group (*n* = 17)	Non-sensitive Group (*n* = 18)	*p* values
Age (SD)			
Child Age	7.65 (1.77)	7.72 (1.45)	0.504
Parent Age	38.59 (3.73)	39.11 (3.95)	0.333
Child Sex (%)^†^			0.127
Male	11 (65%)	7 (39%)	
Female	6 (35%)	11 (61%)	
Race/Ethnicity (%)^†^			
Black/Hispanic	2 (12%)	0 (0%)	0.134
Black/Non-Hispanic	3 (18%)	0 (0%)	0.176
White/Hispanic	0 (0%)	0 (0%)	n/a
White/Non-Hispanic	10 (59%)	16 (89%)	0.013*
Other/Multiple	2 (12%)	2 (11%)	0.952
Location (%)^†^			
Rural	4 (24%)	3 (17%)	0.612
Urban	6 (35%)	3 (17%)	0.208
Suburban	7 (41%)	12 (66%)	0.130
Family Size	2.57 (1.00)	2.16 (0.92)	0.200

Percent or standard deviations in parentheses. Groups compared using Wilcoxson’s ranksum test due to unequal distribution unless otherwise specified.

^†^Chi-squared test for categorical variables.

* *p* < .05.

### 2.2 Outcome measures and data collection

The wrist-worn ActiGraph GT9X (ActiGraph Corp, LLC, Pensacola, FL) was used to collect motor activity using an internal accelerometer, gyroscope, and magnetometer. Actigraphy data collection was set at a sampling rate of 30 Hz to collect data with 60 s epoch lengths, collected continuously across 2 weeks. Additionally, caregivers completed a morning and evening sleep diary for the two-weeks of data collection. Sleep diary questions of interest in this analysis included the timing of (i) the start of the settling down and (ii) perceived sleep onset. For this secondary data analysis, we included participants who had actigraphy and completed sleep diary entries for at least four evenings/nights to ensure that sufficient data was available for the analysis of the child’s settling down experience.

A total of 227 evenings of data were collected from the SS group with an average of 13.33 evenings per participant (SD = 2.44). Children in the NSS group provided a total of 203 evenings of data with an average of 11.27 evenings per participant (SD = 2.85).

### 2.3 Actigraph dataset and preprocessing

Raw actigraphy data was processed through ActiLife (Actigraph Corp.), the accompanying proprietary software of the device. Vector magnitude, or activity counts, per minute were extracted for each participant. Sleep periods were first isolated using the Tudor-Locke (Tudor-Locke et al., 2015) algorithm that labeled each minute as “wake” or “sleep”. Then, the study team used a visual inspection and the caregiver reported sleep onset/offset timing within the sleep diary to adjust the identified sleep periods as needed. In the event that the caregiver reported timing different by 30+ minutes, the algorithm-identified time and visual inspection was prioritized. This prioritization was chosen *a prioi* based on the recommendations from the SBSM Guide to Actigraphy Monitoring (Ancoli-Israel et al., 2015) and the possibility that caregivers may not be with their child when they fall asleep and therefore may need to estimate sleep onset timing. After the sleep periods were isolated, every complete sleep period (no off-wrist time) was analyzed using the Sadeh algorithm (Sadeh et al., 1994). The Sadeh algorithm is a pediatric-specific algorithm that derives sleep variables from identified sleep periods. These variables include sleep duration, sleep efficiency, sleep onset and offset, among others. For this analysis, we only used the sleep onset variable to isolate the settling down period prior to the sleep period.

Next, we identified the start of settling down time within the actigraphy data. During the initial data collection, caregivers were interviewed and described their child’s regular bedtime routine. Caregivers were instructed to write down the start of their child’s settling down time each night in the sleep diary. This was operationalized in this way: the time when the bedtime routine had ended and the child is attempting to fall asleep. For this analysis, we extracted (i) the reported start of the settling down period from each sleep diary and (ii) sleep onset time indicated using the Sadeh algorithm with the cleaned actigraphy data (as outlined above). Using these time points, we cut and indexed the actigraphy data files containing three-dimensional accelerometer data and vector magnitude for each night per participant. Data files were labeled by group status (SS or NSS). Average settling down length of time was assessed using Hedges’ g effect size calculations, where g > 0.41 indicates minimal practical significance. (Ferguson, 2009). The data were then separated into trials using rolling windows to populate the dataset and to create features (see *Classification results* section below). Then, features were extracted from all of the trials. The features were picked to specifically demonstrate power and variation within the time windows and characterize some clinically meaningful aspects of the time period (measures of activity magnitude, variability of movement from moment to moment). These features were mean magnitude of activity, maximum magnitude, kurtosis, skewness, Shannon entropy (Shannon, 1948), standard deviation, and interquartile range (definitions and equations are found in the Supplementary Appendix).

### 2.4 Data classification and analysis

The main aim of the analysis was to characterize differences in the actigraphy data during the settling down time for children with SS and NSS using supervised learning. We approached this binary classification problem using a 10-fold cross-validation with all of the features extracted from the data (see above paragraph). *k*-fold cross validation is used to test a model built to correctly identify a participant with SS from those with NSS across the entire dataset (called a *binary classification problem*). This approach allowed us to observe whether the approach, the dataset, and the method correctly separate the two groups in the dataset with sensitivity, specificity, and accuracy.

First, we aimed to examine how different algorithms performed the binary classification problem. We trialed random forests, support vector machines, linear and quadratic discriminant analyses, Naïve Bayes classification, and a multilayer perceptron model. Random forests performed the best as evidenced by the highest values of the average area under receiving operator characteristics curve (AUC). This finding indicates that sensitivity (identifying true positives) and specificity (identifying true negatives) were both high. Random forests are a collection of decision tree estimators used for regression and classification, where the decision trees are fit with subsamples of the entire dataset (Breiman, 2001). It is a type of supervised machine learning, where a model is trained with labels (SS or NSS). Training and test datasets are created from the entire dataset using a 4:1 split. The best parameter set for the task is found using a grid search algorithm within the training dataset using cross-validation. The parameters searched were the number of decision trees and maximum depth. The end-result was decided based on majority voting of decision trees.

Rolling windows were used to ‘epoch’ data entries into data windows and features were created from the windowed data. Rolling windows are widely used for feature engineering in machine learning algorithms as a robust approach to populate dataset and create more meaningful features. Data from each settling down time were examined in smaller sections (ranging from 7 to 13 min, see Table 2) and features like maximum magnitude were extracted. Then that “window” was moved down (“stride”) either 1 or 3 min and the new window of data was analyzed. Using rolling windows allowed for patterns to be detected within shorter time spans (small windows) rather than compressing the whole settling down period into a single trial (like averaging across the whole night).

**TABLE 2:** Comparison of window and stride lengths using 10-fold cross-validation with random forests.

Window-stride	AUC
7-1	0.82
7-3	0.66
9-1	0.87
9-3	0.71
**11-1**	**0.92**
11-3	0.74
13-1	0.82
13-3	0.69

Bolded text indicates the highest AUC and chosen window and stride length.

First, the relation between window/stride-lengths of data windows and classification performance was examined to see if there is any specific correlation with lengths and performance metrics in a 10-fold cross validation analysis. Window length determines how long the data window will be in minutes, where stride determines the time difference between consecutive windows.

Next, the pair that resulted in the best performance was picked to do in-depth 10-fold stratified cross-validation analysis following the same method. Stratification was used to maintain the ratio between different labels—indicating whether the data came from a participant with SS or not—while also ensuring a balanced distribution of data across different subjects. Cross-validation was done to ensure the model was generalizable for our problem, and stratification was used to ensure uniform data imbalance across different folds. Within each cross-validation fold, a random-forest model with previously identified optimal parameters was trained with that fold’s training set and tested with the corresponding test set. Setting a parameter set instead of finding optimal parameters for each specific fold was chosen to have a more generalizable model. Based on predicted training labels, the decision threshold was changed from Prob >0.5, which is the default case for binary classification, to a threshold found by Youden’s J statistic, *J = TPR-FPR* where *TPR* is true positive rate and *FPR* is false positive rate based on training data’s ROC curve. This was done to make guesses based on the information from training data.

Finally, we conducted individual simple regression analyses to explore how clinically relevant features (namely, measures of magnitude, duration, and variance of activity) contributed to the variance in settling down length of time, a variable found to be significantly different in our previous work (Hartman et al., 2023). We calculated averages of each child’s activity magnitude, maximum activity magnitude, and settling down duration (in minutes) across all settling down periods. We also calculated median entropy and the standard deviation of the settling down duration as measures of variation. Using the average duration of settling down time as the dependent variable, we used each of the calculated variables above as predictors and controlled for child age and sex. As the sample size was small, we used this analysis to explore potential key measurement and intervention targets that may be substantially impacting settling down duration for children with SS.

## 3 Results

### 3.1 Demographics and settling down lengths

Participants were similar in child age and sex, parent age, community type (urban, suburban, or rural) and family size (Table 1). The groups differed on race and ethnicity, with the SS group being slightly more diverse than the NSS group.

Using the procedure outlined in the previous section, settling down periods were measured for each group. On average, children with SS took 52.63 min (*SD* = 38.31 min) to settle down and fall asleep. This differed from the children with NSS, who took on average 26.19 min (*SD* = 14.20 min) to settle down and fall asleep (*p* = 0.075, Hedges’*g* = 0.95).

### 3.2 Classification analysis

#### 3.2.1 Classification results

Window/stride-length pair analysis results are shown in Table 2. Strides were picked to be either 1 or 3 min, and window lengths were started from 7 min and increased by 2 min until the performance started deteriorating. It was found that 11-min window, 1-min stride pair had the best results in terms of AUC.

As this is a secondary analysis of an observational study it was deemed important to create time windows to compensate for both inherent noise spikes in actigraphy data and non-precise sleep diary recordings. The classification results with 11-min window, 1-min stride pair are given in Table 3. On average, the classifier was able to achieve 84.1% accuracy, 84.4% sensitivity and 82.7% specificity. Here, as data from subjects with SS are denoted as positive class, sensitivity provides information on how correctly the model was able to predict if the data is coming from a subject with SS.

**TABLE 3:** Comparison of classification methods on 11-min 1-stride window data.

Cross-validation Fold	Accuracy	Sensitivity	Specificity
Fold 1	84%	86%	77%
Fold 2	88%	88%	86%
Fold 3	83%	81%	87%
Fold 4	83%	83%	84%
Fold 5	84%	85%	82%
Fold 6	83%	84%	81%
Fold 7	82%	83%	77%
Fold 8	85%	84%	88%
Fold 9	83%	82%	84%
Fold 10	81%	83%	77%
Average	83%	84%	82%

#### 3.2.2 Significant predictors

**FIGURE 1:**
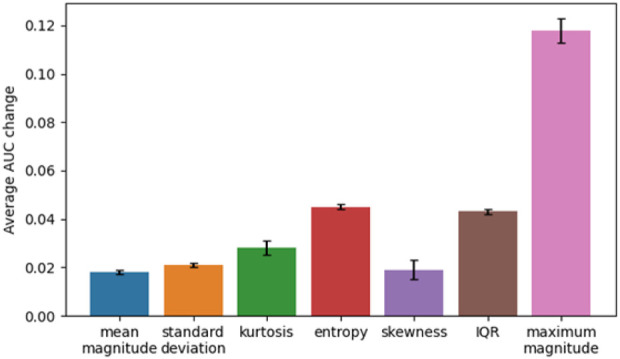
Average AUC change for features.

We also conducted permutation importance testing to determine feature importance in classification of the groups. The features were shuffled 10 times across samples and the model was refitted from scratch to estimate their respective importance. Significant features were mean maximum magnitude of activity, the standard deviation of length of settling down time, and median entropy of activity (Table 4). To understand the magnitude of importance for each feature, we used AUC as the scoring metric. In Figure 1, we show the graph where all features are presented with their respective average AUC decrease. The greater the AUC decrease was when a feature is removed, the more informative that feature was for the classification task. Although the permutation importance test did not show any reason to omit any of the features, maximum magnitude was the most important feature and standard deviation of activity, entropy, and interquartile range were the important features that represent the variability of the window. We chose these most informative features to feed into the *post hoc* regression feature set.

**TABLE 4:** Significant predictors of group using actigraphy data during the settling down time.

Variable	Sensory Sensitive Group (*n* = 16) *M (SD)*	Non-Sensitive Group (*n* = 17) *M (SD)*	*U*	*p*
Mean settling down length of time	72.89 (52.17)	33.11 (13.09)	-1.12	0.264
Mean magnitude	2481.27 (1004.46)	1964.06 (779.16)	-1.55	0.121
Mean maximum magnitude	10663.81 (4924.01)	7347.41 (2086.77)	-2.23	0.026*
Standard deviation of settling down time (mins)	27.05 (14.16)	19.74 (8.23)	-2.63	0.008*
Median entropy	3.43 (0.80)	2.67 (0.72)	-2.34	0.019*

Mann-Whitney *U* test used to compare groups.

*p < 0.05.

### 3.3 Post-hoc regression

We conducted multiple simple regressions as exploratory analyses to examine how the identified features contribute to the variance noted in the average duration of settling down time between groups. Regression analyses indicated that mean maximum magnitude, median entropy, and mean magnitude independently accounted for 84%, 73%, and 57% of the variance in settling down length, respectively (Table 5). This suggests that these variables, constructed from features that successfully separate the two groups, are key contributors to the variance seen in settling down lengths between groups.

**TABLE 5:** Standardized regression coefficients predicting mean setting down time (mins).

Variables	Model 1	Model 2	Model 3	Model 4	Model 5
Group	0.48** (13.06)	0.34* (11.19)	0.13 (7.31)	0.49** (14.66)	0.13 (9.56)
Mean Magnitude		0.62*** (0.006)			
Mean Maximum Magnitude			0.08*** (0.001)		
Standard deviation of settling down				0.300 (0.61)	
Median Entropy					0.79*** (5.68)
Constant (non-standardized *b*)	22.55 (35.29)	-62.98 (33.96)	-43.94* (18.29)	19.67 (36.25)	-79.34** (26.17)
Observations	33	33	33	33	33
R-squared	0.272	0.566	0.837	0.278	0.734
F-test	3.604	9.132	35.90	2.692	19.30
Prob > F	.025*	<.001***	<.001***	.051	<.001***

All regression models controlled for age, sex. Standardized beta coefficients presented with standard error in parentheses.

***p < 0.001, **p < 0.01, *p < 0.05.

## 4 Discussion

Difficulties within the settling down period is often a main contributor to reported sleep problems for children. Current measurement of settling down difficulties rely on caregiver-reported questionnaires. However, school-aged children may not have their caregiver in the room while they fall asleep. Current actigraphy-based measures of sleep do not include characterizing the settling down period specifically and can sometimes miss impactful difficulties. This secondary data analysis provides a novel way to examine activity in the settling down period using actigraphy and sleep diary data and machine learning techniques.

We used an existing data set with children with SS and NSS and the multidisciplinary expertise of our team (occupational therapy, engineering, psychology) to develop a novel way to isolate and characterize differences in settling down time. We chose this data set specifically because, prior to this analysis, we had identified that children with SS were reported to take longer to fall asleep than children with NSS, per caregiver report (Hartman et al., 2023). In the original study, traditional actigraphy sleep variables did not highlight differences between children with SS and NSS, however caregivers were reporting through questionnaires and qualitative interviews significant and impactful sleep problems for children with SS (Hartman et al., 2022). We identified that the settling down period needed further analysis to understand these differences. We chose machine learning because it was a less complex process that may be feasible for sleep science to incorporate in future research.

Machine learning techniques can provide opportunities to examine large amounts of time-series data in ways that elevate outcome measurement analysis to more specifically inform and evaluate intervention changes (Gurchiek et al., 2019; Linden and Yarnold, 2018). This is especially pertinent as sleep interventions are adapted for unique pediatric populations. We found a machine learning approach that was sensitive to the challenges caregivers of children with SS were strongly endorsing (i.e., sleep resistance behaviors, extended sleep onset latency (Hartman et al., 2022; Lufi and Tzischinsky, 2014; Mimouni-Bloch et al., 2021; Manelis-Baram et al., 2021)) but were not seen in traditional sleep variables from actigraphy. Moreover, we found that a less-complex, potentially more accessible random forest classifier best described the pattern of our dataset and was able to successfully distinguish between the two groups with accuracy.

Machine learning classification was able to feasibly discriminate between children with SS and NSS using a small portion of actigraphy data and time-domain features. This finding suggests that perhaps differences in settling down time at night may help us identify children with SS. Sleep and sensory processing differences have been found to be linked for school-aged children using caregiver-reported questionnaires (Hartman et al., 2022; Raj et al., 2024; Lane et al., 2022). Actigraphy data could provide a more objective measure of behavioral outcomes (activity during bedtime in this instance) that differ for children with SS and NSS.

The features we used in this analysis were chosen to describe different clinically relevant characteristics of the data and mainly aimed to explain variation in activity across the settling down period. In this analysis, we identified three key features of the settling down period that can differentiate children with SS and NSS: mean maximum magnitude, median entropy, and mean magnitude. Clinically, these features suggest that children with SS have greater, more frequent, and more variable activity during the settling down period than peers with NSS. This could be related to a number of aspects that have been identified to impact children with SS: increased discomfort due to tactile sensitivities (Tzischinsky et al., 2018), increased arousal levels at bedtime (Mazurek and Petroski, 2015), movement related to parent involvement in the settling down (e.g., laying with their child) (Meltzer and Montgomery-Downs, 2011).

Existing clinical sleep interventions recognize the importance of decreasing “activity magnitude and variability”, or overall activity and energy level, to support improved sleep health by incorporating a calming and consistent routine, dimming lights, and providing children with predictable indicators that they should be settling down (e.g., taking a bath or reading a book) (Hornsey et al., 2024). It could be that children with SS benefit from this type of intervention specifically or need more intense focus on decreasing activity during the settling down time. Future research could test these hypotheses. Future studies are needed to probe this further.

In this analysis, we used time periods informed by caregiver reported settling down start times. However, we also trialed cutting the actigraphy data into segments based solely off actigraphy data to test a method that could be applied when sleep diaries are not available. We tried setting the start of settling down time 60 min prior to actigraphy derived sleep onset which produced a classifier that performed worse with 0.67 AUC overall. This exploration highlights the importance of gathering caregiver reported settling down start time for optimal performance using this technique.

Finally, our *post hoc* regression analysis indicated that activity during the settling down period, specifically the intensity (maximum magnitude) and variance (median entropy), are important behavioral outcomes specific to children with SS. While it is unsurprising that our predictors are significant within our model, considering they were derived from the features that best separated the groups, it is interesting to consider the amount of variance each predictor accounts for in settling down duration (*R* ^*2*^). Considering that this study was observational, we do not know if these activity-related variables are the cause of the difficulties settling down for children with SS. Future research can test, in a causal way, the impact of different interventions on sleep and sensory-related behavior outcomes that might not be supportive of sleep (e.g., maximum magnitude or a lot of large movements during setting down).

### 4.1 Strengths and limitations

To our knowledge, this paper is the first to present application of machine learning techniques to examine the settling down period of activity for a pediatric sample. We used several techniques to strengthen the analysis. Time windows were used to compensate for inherent noise spikes in the actigraphy data and non-precise sleep diary recordings. 10-fold cross-validation was used to address concerns of bias related to random forest analysis. Despite these strengths, limitations remain. Most importantly, the small sample size and the semi-controlled nature of this study resulted in limited power to understand the significance and magnitude of the contribution each predictor had on the variance of settling down duration. Within this small sample, we did have baseline differences in racial and ethnic diversity, with the SS group being slightly more diverse than the NSS group. This could be reflective of a true difference between children with have SS and those without, however, our sample was too small to comment further. Both groups are not extremely diverse and are reflective of the typical sample of families that engage in research in the Pittsburgh area through a research registry.

Additionally, generalizing beyond the sample within this study is difficult due to lack of diversity and breadth of sampling. Future studies with larger, more generalizable samples are needed to better understand the differences during the settling down period for children with sensory sensitivities compared to peers without sensitivities. As a secondary data analysis, this paper is limited to the data originally collected. As such, we relied on caregiver-reported start of settling down to indicate where to cut the actigraphy data. Future research in this area may consider using an event marker on the actigraphy device or a more precise description of what settling down means to the caregiver-child dyad.

Also, the best parameter set was found using cross-validation based on the results acquired from an unobserved portion. The 10-fold cross-validation results given in Table 3 were acquired using the same portions used in the hyperparameter tuning. This may create a slightly optimistic bias in the analyses, but this was done due to the limited dataset size. Future research in this area should include more data collection from a more diverse population, and create a more generalizable approach by separating a portion of the dataset specifically for validating the machine learning approaches’ success.

### 4.2 Future directions

We believe that the settling down period will continue to be an important period to examine for future sleep research. We aim to provide a novel way to characterize the settling down period for future studies to test within their own, hopefully larger, samples. A future study to test this current analysis with a larger sample of children with SS and NSS would further develop a foundation upon which this line of research can grow. Additionally, we suggest that future research confirm the increased and variable activity during the settling down period for children with SS and explore potential causes. Additionally, interventions targeting the settling down period can use this machine learning approach to measure change or characterize targets for specific pediatric populations who have unique barriers to good sleep health. Using machine learning techniques may allow for the settling down period to be analyzed in depth to see if it can be predicted solely by looking at actigraphy data and its extracted features.

## Data Availability

The raw data supporting the conclusions of this article will be made available by the authors, without undue reservation.
