# Supplementary material for: What goes on when the lights go off? Using machine learning techniques to characterize a child’s settling down period
Source: Front Netw Physiol. 2025 May 30;5:1519407. doi: 10.3389/fnetp.2025.1519407 (PMC12162617; doi:10.3389/fnetp.2025.1519407)
Supplement: Supplementary file 1 [file Supplementaryfile1.docx]

# Appendix

Extracted features of interest

| **Feature** | **Equation** | **Explanation** |
| --- | --- | --- |
| Mean magnitude | $\mu_{n}=\frac{1}{N}\sum n_{i}$ | To capture average behavior of a time window. |
| Maximum magnitude | $mag_{max}=max(n_{i})$ | To capture the highest activity in a time window. |
| Kurtosis | $K\left( n \right)=\frac{\mu_{n}^{4}}{\sigma^{4}}$ | “Tailedness” of the probability distribution of time window. $\mu_{4}$ is the fourth central moment and $\sigma^{4}$ is variance-squared. |
| Skewness | $\tilde{\mu_{3}}=\frac{\mu_{n}^{3}}{\sigma^{3}}$ | Measures the “tiltedness”, or asymmetry, of the probability distribution of time window. |
| Shannon entropy | $S\left( n \right)= \sum_{j} p_{n}\left( j \right)ln(\frac{1}{p_{n}\left( j \right)} )$ | Computes the amount of uncertainty in the time window. |
| Standard deviation | $Std\left( n \right)=\sigma_{n}=\sqrt{E\left[ n^{2} \right]-E^{2}[n]}$ | Computes amount of variation in the time window, average distance from the mean. |
| Interquartile range | $IQR=Q_{3}-Q_{1}$ | Computes amount of variation in the time window, robust to outliers. Average of squared distances from the mean. |
